# An investigation into the zoning of ecosystem sensitivity control areas in Mentougou District (Beijing, China)

**DOI:** 10.1371/journal.pone.0316025

**Published:** 2024-12-19

**Authors:** Xiaodan Li, Haoyu Tao, Jing Li, Zhen Liu, Zhiping Liu

**Affiliations:** 1 State Key Laboratory for Tunnel Engineering, China University of Mining and Technology (Beijing), Beijing, China; 2 School of Mechanics and Civil Engineering, China University of Mining and Technology (Beijing), Beijing, China; National Institute of Technology, India (Institute of National Importance), INDIA

## Abstract

To achieve a more precise delineation of ecosystem sensitivity control areas, this study examines the Mentougou District in Beijing and introduces the Ecosystem Sensitivity Control Area Classification Method (ESCACM). This novel approach combines single-factor sensitivity analysis, multi-factor comprehensive sensitivity assessments, and strategies for zoning based on various ecological scenarios. The study categorizes the region into three specific zones: first-level management and control areas, second-level management and control areas, and non-management and control areas. The key findings are: (1) By systematically categorizing relevant factors, the method creates independent, non-overlapping groups, effectively reducing dimensionality. (2) After conducting a comprehensive sensitivity assessment using multiple factors, scientifically quantified values are assigned to each zone. These values are processed through mathematical overlay algorithms, which generate composite results with different sensitivity levels. This method places a strong emphasis on quantitative analysis, thereby enhancing the objectivity and precision of the results. (3) The use of multi-scenario ecosystem sensitivity zoning strategies enhances the adaptability and flexibility of the zoning process. This method significantly improves the accuracy and scientific credibility of ecosystem sensitivity zoning, providing a versatile approach to meet the varied zoning needs of different regions. This model serves as a valuable framework for promoting ecological protection efforts in support of sustainable urban development objectives.

## 1 Introduction

With evolution of the socio-economic landscape, ecological and environmental challenges have become increasingly significant [1], emerging as crucial topics demanding attention. In this context, assessing ecosystem sensitivity is vital for guiding environmental protection, promoting sustainable development, and enabling the scientific planning of regional development strategies [2]. Precisely delineating ecosystem sensitivity control areas is a key tool for directing socio-economic development effectively, ensuring sustainability for both the ecology and the environment [3, 4].

Ecosystem sensitivity reflects how an ecosystem within a region responds to human activities or natural changes, indicating the probability and ease of regional ecological issues arising [5]. It acts as a crucial marker for evaluating potential outcomes from external disturbances. As an important tool, assessing ecosystem sensitivity aims to identify areas most at risk of environmental degradation and those of highest conservation value, offering a scientific foundation for the strategic delineation of ecological function zones. The evaluation mainly focuses on critical ecological factors such as tidal wetlands [6, 7], nature reserves, river systems [8], mountainous and hilly terrains [9], ecological fragility [10], and forest vegetation [11].

Based on ecosystem sensitivity assessments, zoning research attempts to classify regions into different sensitivity levels, allowing for targeted conservation efforts. However, existing zoning methods are somewhat simplistic, primarily utilizing the Analytic Hierarchy Process (AHP) to assign weights to each indicator factor. These weights inform a weighted overlay analysis on a Geographic Information System (GIS) platform, creating a comprehensive ecosystem sensitivity evaluation map. This map is then divided into regions using the natural breaks (Jenks) classification method. This approach is prevalent across various applications, including zoning of ecologically sensitive areas [12], assessing ecosystem services in hilly regions [9], urban ecological redlining [13], landscape sensitivity analysis [14], urban land-use suitability [15], development path exploration for hilly cities [16], and coastal marine ecosystem sensitivity assessments [5].

While the method’s straightforward design allows for considering multiple factors and their relative weights, making it practical for use, it also presents notable limitations: (1) Ecosystem sensitivity research involves numerous interconnected factors that may contain overlapping information, potentially compromising assessment accuracy. (2) The process relies heavily on subjective weight assignment via AHP, leading to qualitative over quantitative analysis due to subjective judgment, which may detract from decision-making rigor and precision. Consequently, there is an urgent need to develop a more objective and scientifically robust approach for ecosystem sensitivity zoning. (3) Under varied regional development conditions and policy directives, the method’s lack of capacity for differentiated zoning strategies limits its adaptability and flexibility.

As one of China’s most economically advanced and densely populated areas, Beijing has faced mounting ecological challenges such as environmental degradation, increased soil erosion, and severe air pollution [17]. Mentougou District, located in Beijing’s western ecological conservation zone, is vital to maintaining the city’s sustainable development. Historically, Mentougou was one of China’s primary anthracite coal production areas, with coalfields covering approximately 500 km^2^, serving as a crucial energy source for the capital [18]. While coal mining spurred economic growth, it left lasting ecological damage. These impacts manifest as surface subsidence, landslides [19], vegetation degradation, soil contamination, and water pollution, contributing to a range of environmental challenges. The landscape structure has undergone significant transformation, marked by reduced landscape connectivity and increased heterogeneity, reflecting a progressive trend toward fragmentation [20]. Between 2010 and 2015, Beijing saw significant changes in vegetation cover compared to the previous five years. Notably, areas experiencing vegetation recovery accounted for 12.81%, indicating positive results from ecological restoration efforts. However, 9.49% of areas experienced vegetation degradation, predominantly concentrated in Mentougou District [21]. This concentration highlights the urgency and importance of addressing the district’s environmental issues.

To effectively curb further ecological degradation, this study takes Beijing’s Mentougou District as a case study and proposes an innovative Ecosystem Sensitivity Control Area Classification Method (ESCACM). This approach divides the study area into three tiers: the first-level management and control area (FMCA), designated as the core ecological protection zone, where the strictest conservation measures are enforced, fully prohibiting any form of development or construction; the second-level management and control area (SMCA), which implements differentiated control measures, banning any development that may impair ecosystem functions; and the non-management and control area (NMCA), identified as a suitable zone for urban development, possessing strong resilience to external disturbances and allowing orderly construction activities aligned with urban planning policies.

This offers several distinct advantages. First, it strategically reduces factor dimensionality by scientifically organizing related factors into new, independent, and non-overlapping categories, thereby simplifying theoretical complexity. Second, after conducting a multi-factor comprehensive sensitivity assessment, each sensitivity zone is assigned scientifically quantified values. These values are processed through mathematical overlay algorithms, resulting in composite outcomes with varying sensitivity scores. This approach enhances the emphasis on quantitative analysis, ensuring greater objectivity and precision in the final outcomes. Third, the use of multi-scenario ecosystem sensitivity zoning strategies allows the method to flexibly adapt to diverse regional development needs and policy directives, greatly improving the adaptability and suitability of the zoning process.

The key contributions of this study are highlighted in several areas: (1) It introduces a novel approach to ecosystem sensitivity zoning, which significantly enhances the precision and scientific validity of the analysis. (2) The use of multi-scenario ecosystem sensitivity zoning strategies greatly improves the adaptability and flexibility of spatial planning, allowing for dynamic adjustments in response to local conditions. This approach promotes a balanced and synergistic relationship between ecological conservation and economic growth, thereby fostering long-term sustainability.

## 2 Materials and methods

### 2.1 Study area overview

**Fig 1:**
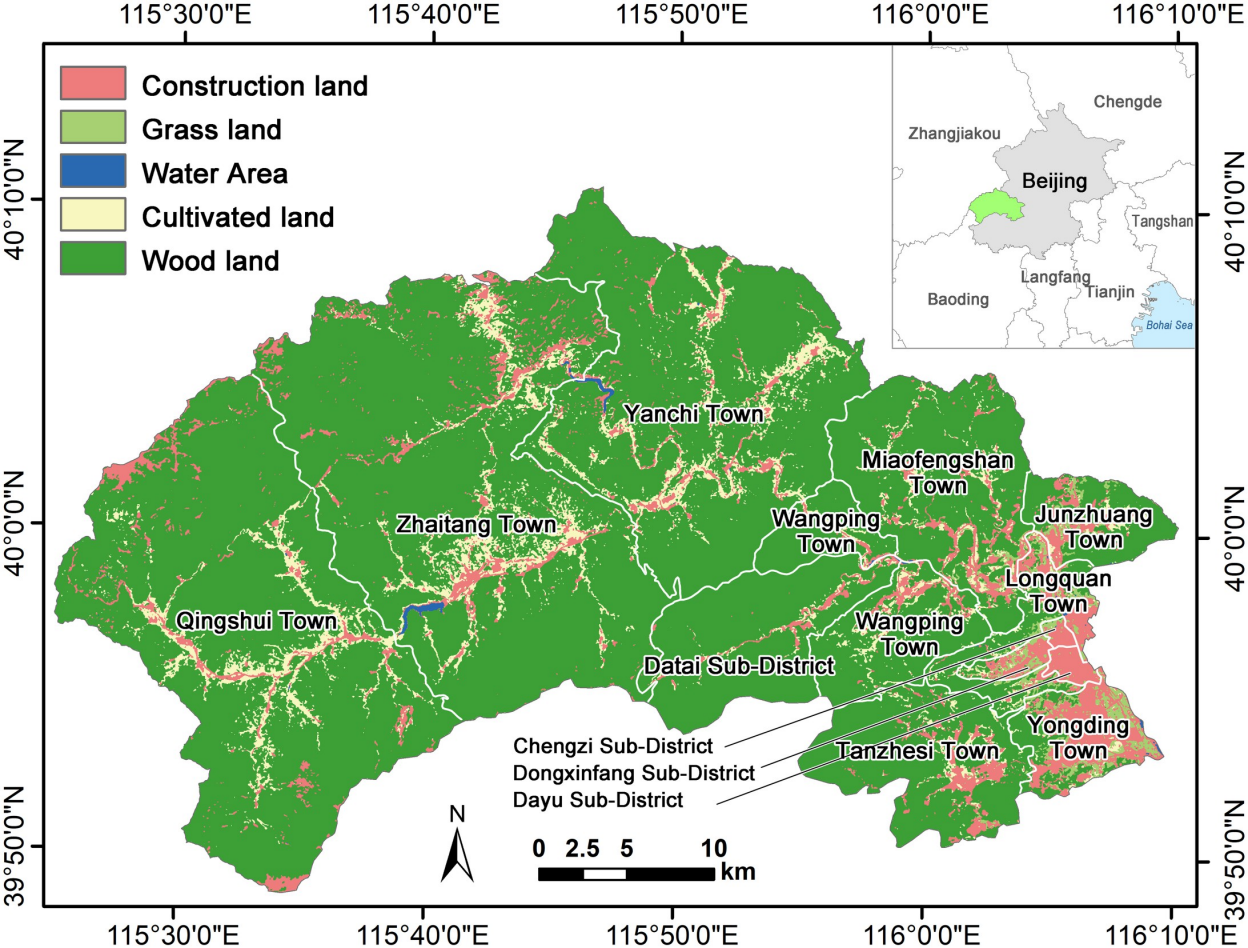
Geographical location of the Mentougou District.

The Mentougou District is located southwest of Beijing city, at coordinates 115°25′0′′-116°10′9′′E and 39°48′41′′-40°10′39′′N (Fig 1). It extends approximately 62 km from east to west and 34 km from north to south, covering a total area of 1,448 km^2^. As of 2022, the district had a permanent population of 396,000, with a moderate population density of 273 people per square kilometer. Renowned for its abundant natural resources, Mentougou features rugged mountains, dense forests, and a well-developed network of water bodies, creating a uniquely favorable ecological environment. The terrain is mainly mountainous, with the mountainous area accounting for about 98.5% of the total area of the district. The forested area is 1210 km^2^, accounting for about 84% of the total area of the district. The vegetation is mainly natural woodland, covering a wide area, and is rich in species. The distribution of vegetation varies considerably according to the altitude and slope aspect of the terrain; there are some cultivated lands and grasslands in the valley and flat areas, accounting only for a small total area. There are more than 300 large and small tributaries in the area, and there are abundant water systems. The large rivers include the Yongding River, Qingshui River, and Baigou River. The Mentougou District is a famous historical and cultural touristic and leisure area with scenic tourist attractions such as the Baihuashan National Nature Reserve, the Nanshiyang Grand Canyon Forest Park, the Shuanglongxia East Mountain Forest Park, and the Tanzhe and Jietai Temples.

### 2.2 Data sources

The foundational data for this study includes various datasets such as Digital Elevation Model (DEM) data, water source protection areas, Landsat-8 remote sensing imagery, precipitation levels, soil erosion intensity, soil heavy metals, soil pH alkalinity, national nature reserves, scenic areas, and the nighttime light index. The detailed sources of these datasets are provided in Table 1.

**Table 1:** Data description and sources.

Data name	Resolution	Sources
**DEM, Landsat-8 remote sensing image**	30 m	https://www.gscloud.cn
**Water source protection areas**	30 m	http://www.beijing.gov.cn
**Precipitation, Soil erosion intensity, Soil heavy metals, Soil pH alkalinity**	30 m	http://www.gisrs.cn
**Water areas, Road**	30 m	https://www.rivermap.cn
**National nature reserves, Scenic areas, Cultural heritage**	30 m	The Mentougou Zoning Plan (Territorial Spatial Planning) (2017–2035) and The Fangshan Zoning Plan (Territorial Spatial Planning) (2017–2035)
**Nighttime light index**	130 m	http://www.hbeos.org.cn/
**Coal mine database**	30 m	http://ngac.org.cn/Kuangchandi/index.html

### 2.3 The principles of ESCACM

The ESCACM is a differentiated zoning strategy carefully crafted considering the region’s current developmental context and anticipated governmental directives. Its objective is to manage and protect ecosystem sensitivity efficiently by accurately delineating control areas. The operational principles are divided into three components: ecosystem single-factor sensitivity analysis, multi-factor comprehensive sensitivity assessments, and a multi-scenario ecosystem sensitivity zoning strategy.

In the first part, Ecosystem Single-factor Sensitivity Analysis, the study categorizes 18 ecosystem sensitivity factors into topographic and geomorphological factors, ecological environmental factors, and human activity factors. Using GIS technology, a sensitivity analysis is conducted for each category.

The second component involves Multi-factor Comprehensive Sensitivity Assessments, where the AHP is used to determine the weight of each factor within its category. A weighted overlay analysis is then performed using the ArcGIS platform, resulting in a comprehensive sensitivity map. Areas are classified through the natural breaks (Jenks) method into highly, moderately, and non-sensitive zones, with respective values of "3," "2," and "1.

The final component, the Multi-scenario Ecosystem Sensitivity Zoning Strategy, involves rasterizing and summing the three comprehensive sensitivity maps using the ArcGIS platform, producing grid values ranging from "3" to "9." These values are then classified into three scenario types: priority ecological protection, balanced ecological and construction development, and development-oriented construction.

**Fig 2:**
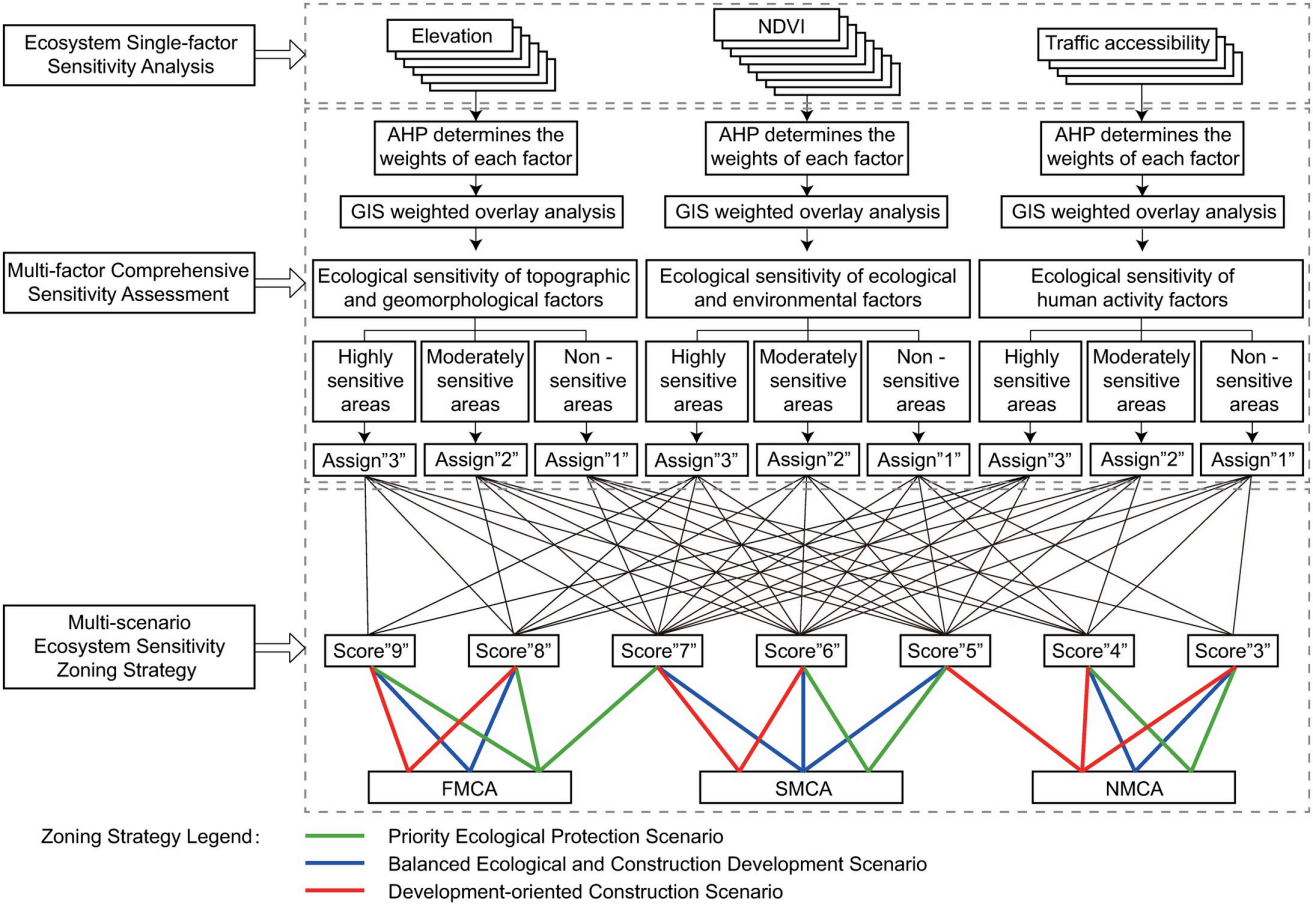
Technical flow chart of ESCACM.

1. **Priority Ecological Protection Scenario:** This scenario emphasizes preserving the ecosystem’s integrity and stability, with strict controls on land development. Areas with scores of "7," "8," and "9" are designated as FMCA, scores of "5" and "6" as SMCA, and scores of "3" and "4" as NMCA.
2. **Balanced Ecological and Construction Development Scenario:** This scenario seeks a balance between ecological protection and economic development. Here, areas with scores of "8" and "9" are designated as FMCA, scores of "5," "6," and "7" as SMCA, and scores of "3" and "4" as NMCA.
3. **Development-oriented Construction Scenario:** This scenario prioritizes construction and development, ensuring that significant pollution is avoided. In this setup, areas with scores of "8" and "9" are designated as FMCA, scores of "6" and "7" as SMCA, and scores of "3," "4," and "5" as NMCA, as illustrated in Fig 2.

## 3 Results

### 3.1 Ecosystem single-factor sensitivity analysis

The selection of ecosystem sensitivity factors aims to reflect distinct and differentiated characteristics, ensuring that each factor is both relatively independent and irreplaceable. Based on this principle and a thorough review of previous studies, 18 ecosystem sensitivity factors were identified for the study area. These factors are divided into three groups based on their attributes [22–26]:

1. **Topographic and Geomorphological Factors:** This group primarily represents the topography and water resource conditions of the Mentougou District. It includes six ecological factors: elevation [10, 27], slope [28], slope aspect, relief degree of land surface [29], water areas [30], and water source protection areas [31].
2. **Ecological and Environmental Factors:** These factors describe the state of vegetation, precipitation, soil quality, and the presence of nature reserves. The eight factors considered in this category are the Normalized Difference Vegetation Index (NDVI) [32], land use types [23], precipitation [10, 17], soil erosion intensity [3, 30, 31], soil heavy metals [33], soil pH alkalinity [34], national nature reserves, and scenic areas [29].
3. **Human Activity Factors:** This group highlights the impact of human activities, which are dominant influences on ecosystems [21]. It comprises four factors: traffic accessibility [35], nighttime light index [36, 37], cultural heritage, and coal mining resource impact factors.

Each factor undergoes a single-factor sensitivity analysis, with sensitivity levels classified into five categories using the natural breaks (Jenks) classification method in the ArcGIS platform. These categories are extremely sensitive areas, highly sensitive areas, moderately sensitive areas, slightly sensitive areas, and non-sensitive areas [38].

**Fig 3:**
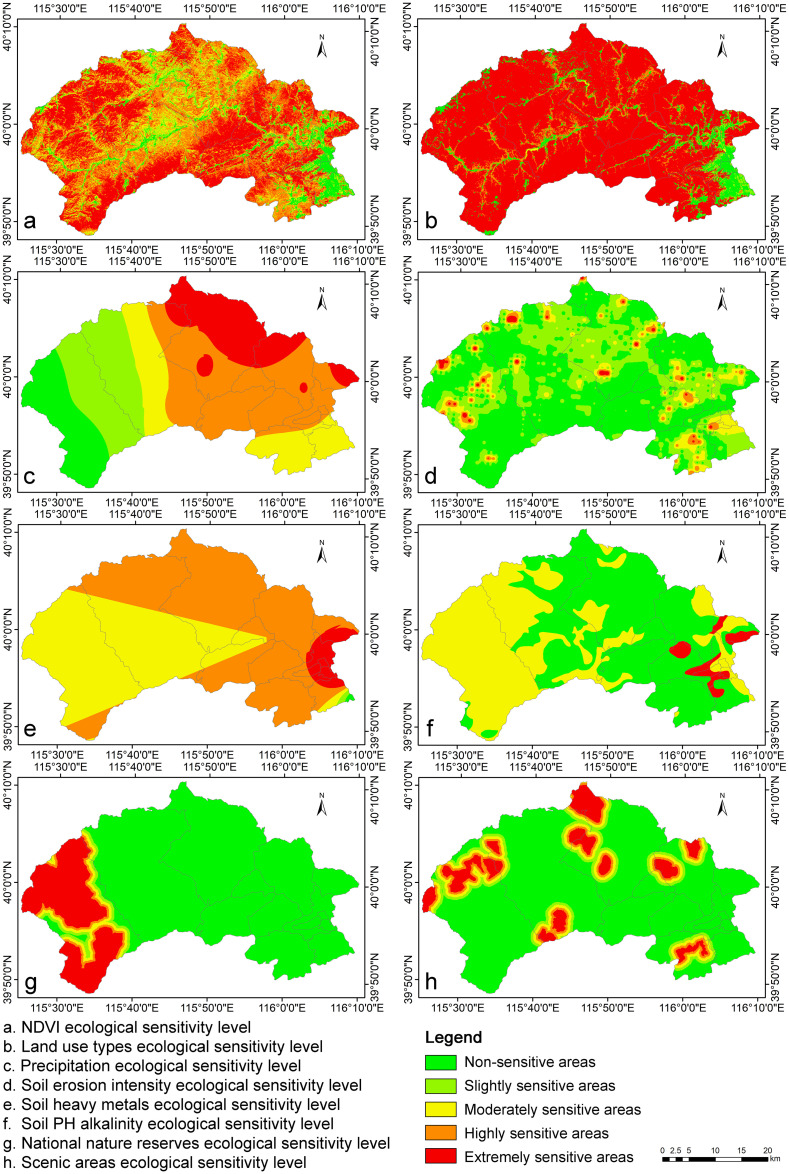
Sensitivity analysis for each individual factor within the ecological and environmental category.

Due to space limitations, this study focuses on the single-factor sensitivity analysis of soil heavy metals as an example. Comprehensive data for additional indicator factors are presented in Table 2. The findings from the single-factor sensitivity analysis pertaining to ecological and environmental factors are illustrated in Fig 3, while the results for topographic and geomorphological factors, as well as human activity factors, can be referenced in S1 and S2 Figs.

**Table 2:** The area and proportion of each indicator factor within the various management areas.

Indicator factors	Extremely sensitive areas		Highly sensitive areas		Moderately sensitive areas		Slightly sensitive areas		Non-sensitive areas		Notes
	Area (km^2^)	Area proportion	Area (km^2^)	Area proportion	Area (km^2^)	Area proportion	Area (km^2^)	Area proportion	Area (km^2^)	Area proportion	
Elevation	131	9%	262	18%	381	26%	423	29%	251	18%	-
Slope	118	8%	295	20%	360	25%	389	27%	286	20%	-
Slope aspect	426	29%	151	11%	347	24%	218	15%	306	21%	Extremely Sensitive Areas: Due South, Southeast; Highly Sensitive Areas: Due East; Moderately Sensitive Areas: Northeast, Southwest; Slightly Sensitive Areas: Due North; Non-Sensitive Areas: Due West and Northwest.
Relief degree of the land surface	39	3%	176	12%	364	25%	498	34%	371	26%	-
Water areas	17	1%	9	1%	9	1%	9	1%	1404	97%	The areas within 30 meters, 60 meters, 90 meters, and 120 meters from the river are designated as Extremely Sensitive, Highly Sensitive, Moderately Sensitive, and Slightly Sensitive areas, respectively.
Water source protection areas	1	0%	1	0%	2	0%	3	0%	1441	100%	The areas within 50 meters, 100 meters, 300 meters, and 600 meters from the water source protection areas are designated as Extremely Sensitive, Highly Sensitive, Moderately Sensitive, and Slightly Sensitive areas, respectively.
NDVI	618	43%	448	31%	189	13%	92	6%	101	7%	-
Land use types	1210	84%	97	7%	3	0%	20	1%	118	8%	The woodland is designated as an Extremely Sensitive area, cultivated land as a Highly Sensitive area, water area as a Moderately Sensitive area, grassland as a Slightly Sensitive area, and construction land as Non-Sensitive areas.
Precipitation	237	16%	490	34%	241	17%	293	20%	187	13%	-
Soil erosion intensity	11	1%	39	2%	114	8%	462	32%	822	57%	-
Soil heavy metals	57	4%	773	53%	613	42%	3	0%	2	0%	-
Soil PH alkalinity	46	3%	-	-	637	44%	-	-	765	53%	Set the pH value ranges “pH < 5 or pH > 8.5,” “5.0 < pH < 6.5 or 7.5 < pH < 8.5,” and “6.5 < pH < 7.5” as Extremely Sensitive, Moderately Sensitive, and Non-Sensitive areas, respectively.
National nature reserves	232	16%	29	2%	33	2%	27	2%	1127	78%	The areas within 100 meters, 500 meters, 1000 meters, and 1500 meters from the national nature reserves are designated as Extremely Sensitive, Highly Sensitive, Moderately Sensitive, and Slightly Sensitive areas, respectively.
Scenic areas	133	9%	71	5%	91	6%	91	6%	1062	74%	The areas within 100 meters, 500 meters, 1000 meters, and 1500 meters from the scenic areas are designated as Extremely Sensitive, Highly Sensitive, Moderately Sensitive, and Slightly Sensitive areas, respectively.
Traffic accessibility	1161	80%	55	4%	62	4%	73	5%	97	7%	The areas within 50 meters, 100 meters, 150 meters, and 200 meters from the road are designated as Non-sensitive, Slightly Sensitive, Moderately Sensitive, Highly Sensitive areas, respectively.
Nighttime light index	1095	76%	306	21%	30	2%	16	1%	1	0%	-
Cultural heritage	1	0%	26	2%	75	5%	109	8%	1237	85%	The areas within 100 meters, 500 meters, 1000 meters, and 1500 meters from the cultural heritage are designated as Extremely Sensitive, Highly Sensitive, Moderately Sensitive, and Slightly Sensitive areas, respectively.
Coal mining resource impact factors	3	0%	65	4%	114	8%	120	8%	1146	80%	The areas within 100 meters, 500 meters, 1000 meters, and 1500 meters from the coal mining site reserves are designated as Extremely Sensitive, Highly Sensitive, Moderately Sensitive, and Slightly Sensitive areas, respectively.

As an important component for human survival and development, the soil is an important part of the terrestrial ecosystem. However, with the accelerated urbanization and mineral resource developments, chemical production and unreasonable use of chemical fertilizers and pesticides have led to the continuous enrichment of heavy metals in the soil. Soil heavy metals are the main soil inorganic pollutants, which are generally not easily dissipated and removed by water and cannot be decomposed by soil microorganisms. Soil heavy metals accumulate in water, plants, and animals [39], resulting in serious environmental pollution [33], thus affecting the entire ecosystem environment.

In this study, we collected data on the soil content of six critical heavy metals, Cd, Cr, Pb, Cu, Zn, and As, in the Mentougou District, Beijing, and applied the potential ecological risk index method proposed by Hakanson for analysis. This method can be used to evaluate the characteristics and environmental behavior of heavy metal pollutants from a sedimentological perspective, which is determined as follows (Eqs 1 and 2, and 3) [40]:

$$C_{f}^{i}=c_{s}^{i}/c_{n}^{i}$$ (1)

where,$C_{f}^{i}$ is the pollution index of an element, and $C_{s}^{i}$ and $C_{n}^{i}$ are the detected concentration and background values of the ith heavy metal element, respectively. The background values of Cd, Cr, Pb, Cu, Zn, and As were 0.119 mg/kg, 18 mg/kg, 24.6 mg/kg, 18.7 mg/kg, 57.5 mg/kg, and 7.09 mg/kg, respectively [41].

$$E_{i}=T_{i}\times C_{f}^{i}$$ (2)

where, *E*_*i*_ is the potential ecological pollution index of a single metal, and *T*_*i*_ is the toxicity response coefficient of a single heavy metal i. The toxicity response coefficient of Cd, Cr, Pb, Cu, Zn, and As were 30, 2, 5, 5, 1, and 10, respectively [40].

$$RI=\sum_{i=1}^{n}E_{i}$$ (3)

where, *RI* is the integrated potential ecological risk index.

According to the ecological sensitivity map of soil heavy metals in the study area (Fig 3e), the soil heavy metal content in the region is generally high. The extremely sensitive and highly sensitive areas are mainly distributed in the eastern and northern areas, with a total area of 830 km^2^, accounting for 57% of the entire region; the moderately sensitive areas are mainly located in the western and central areas, with an area of 613 km^2^, accounting for 42% of the entire region; the slightly sensitive and non-sensitive areas are located in the southeast areas, covering a smaller area of about 5 km^2^.

### 3.2 Multi-factor comprehensive sensitivity assessment of ecosystem

**Fig 4:**
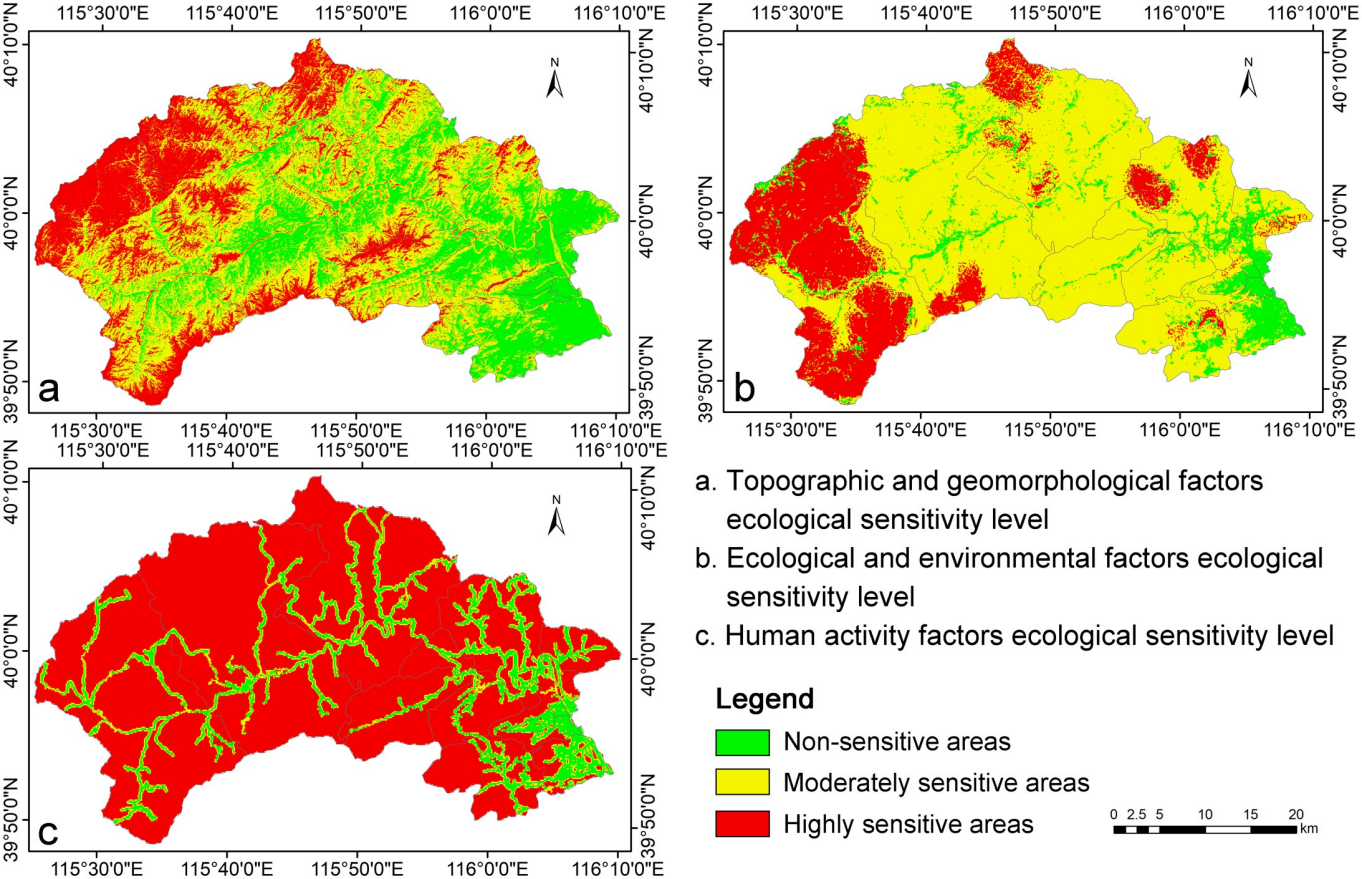
A comprehensive multi-factor sensitivity assessment map of the ecosystem.

Since the AHP [4, 42] and ArcGIS weighted overlay methods are relatively straightforward and user-friendly in practical applications, the detailed steps will not be expanded upon here. By utilizing these methods in conjunction, a comprehensive multi-factor sensitivity assessment map of the ecosystem was successfully generated (Fig 4). For information regarding the specific weight distribution associated with each individual factor evaluation, please see S1–S3 Tables.

### 3.3 Outcomes of the strategic layouts for multi-scenario ecosystem sensitivity control areas

Considering the actual development conditions of the study area and the future planning direction set by the government, this study designates the balanced ecological and construction development scenario as the final optimized layout strategy.

**Fig 5:**
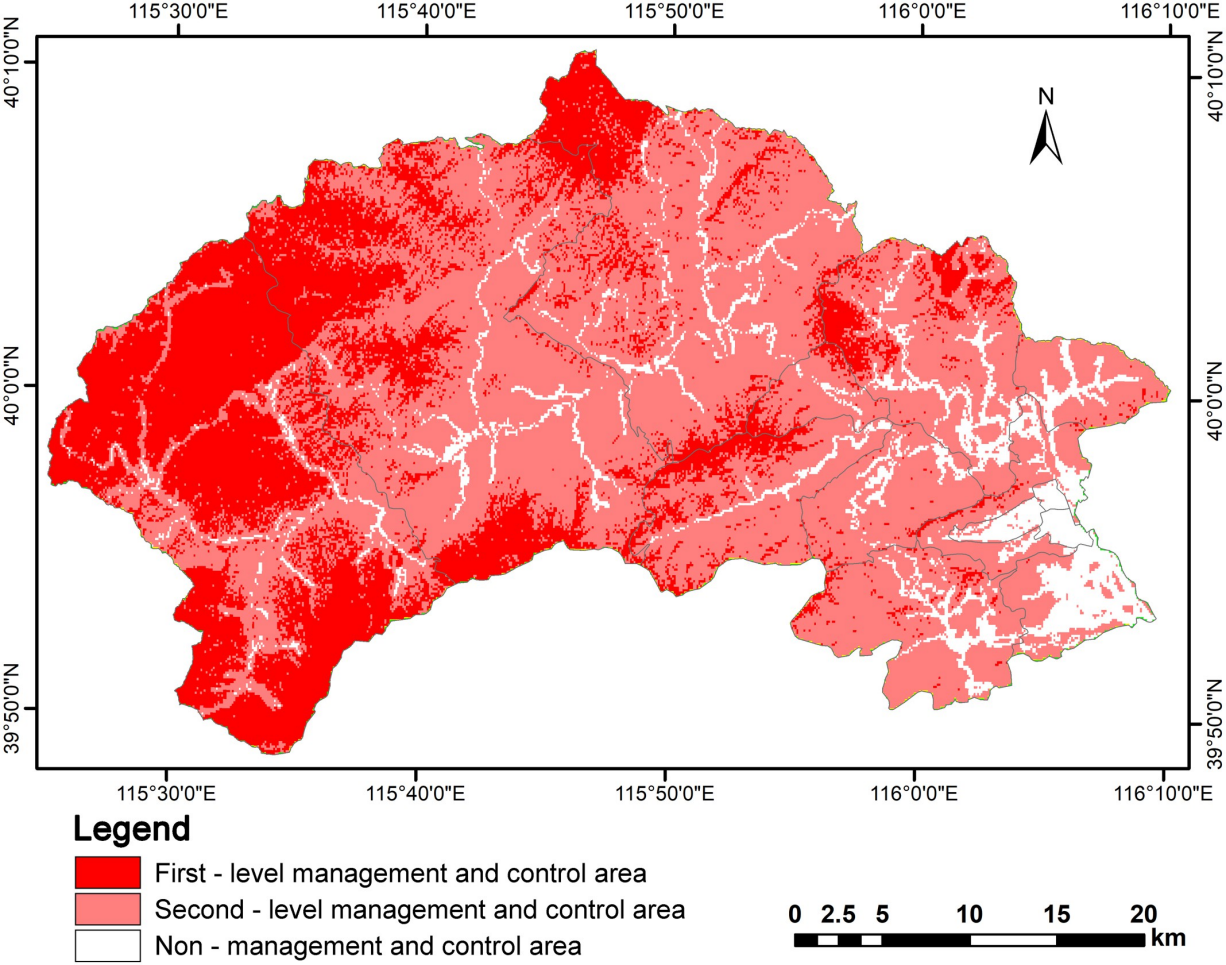
Ecosystem sensitivity control areas zoning map of the Mentougou District.

In the multi-factor comprehensive sensitivity assessment map of the ecosystem, areas classified as highly sensitive, moderately sensitive, and non-sensitive were assigned values of "3," "2," and "1," respectively. Through ArcGIS raster overlay calculations, areas with scores of "8" and "9" were designated as FMCA; areas scoring "5," "6," and "7" were designated as SMCA; and areas with scores of "3" and "4" were designated as NMCA (Fig 5).

The FMCA are primarily situated in the western and central regions of the study area, encompassing locations such as Baihua Mountain National Nature Reserve, Nanshiyang Grand Canyon Forest Park, Shuanglong Gorge East Mountain Forest Park, and Malan Forest Park. These areas cover a total of 450 km^2^, representing approximately 31% of the overall study area. The SMCA predominantly occupy most of the central part of the study area, displaying a more concentrated distribution, with a total area of 880 km^2^, which accounts for about 61% of the total area. In contrast, the NMCA are primarily found in the eastern urban core and along the ring roads in the central part of the study area, covering a total of 118 km^2^, which is roughly 8% of the total study area.

## 4 Discussion

### 4.1 Analysis of the scientific, objective, and systematic advantages of the ESCACM

The traditional classification method involves gathering factors, assigning them corresponding weights, and conducting a weighted overlay, followed by classification using the natural breaks (Jenks) classification method. While the basic principle is relatively simple, it has several significant limitations. To address these issues, this study introduces the ESCACM, which offers two key advantages. First, the method conducts an in-depth analysis of the interrelationships among ecological sensitivity factors, organizing and combining them into independent groups—specifically, topographic and geomorphological factors, ecological and environmental factors, and human activity factors. This classification is significant because it effectively constrains numerous interrelated factors within their respective groups, thereby reducing interference with the overall factor evaluation system and achieving theoretical dimensionality reduction. Second, utilizing the AHP to determine the weight of each ecological sensitivity factor, this method assigns values to the comprehensive sensitivity analysis results for the grouped factors. Subsequently, mathematical methods are employed for raster summation, and the sensitivity levels of the ecosystem are classified according to multi-scenario ecosystem sensitivity zoning strategies. This approach enhances the emphasis on quantitative research, rendering the final results more scientific, objective, and systematic.

**Fig 6:**
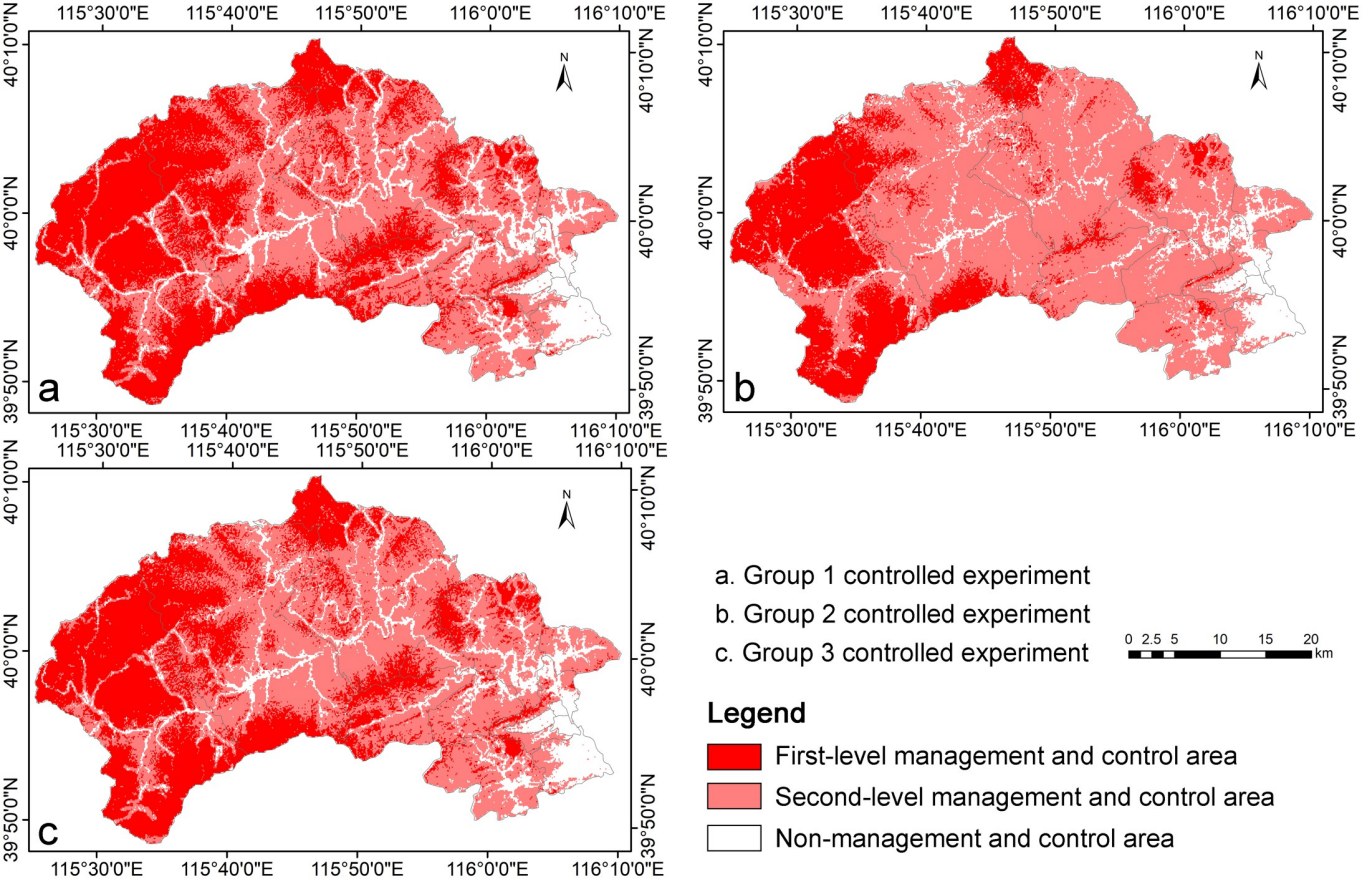
Zoning map of ecosystem sensitivity control areas based on the three sets of controlled experiments.

To comprehensively validate the scientific, objective, and systematic nature of the ESCACM, this study devised three controlled experiments utilizing traditional classification methods. The specific steps of the experimental design are outlined as follows. First, fifteen experts with significant expertise in relevant fields were selected and evenly grouped into three experimental teams, each consisting of five experts, based on the order in which they were invited. Next, each expert independently employed the AHP to determine the weights of topographic and geomorphological factors, ecological and environmental factors, and human activity factors. This approach allowed each expert to assess the importance of each factor objectively, relying on their professional knowledge and experience. Then, the average weight for each factor was calculated within each group (S4 Table), reflecting a consensus on the significance of the factors. This step aimed to minimize the influence of individual differences on the results by harnessing collective judgment, thus enhancing the accuracy and reliability of the findings. Finally, using ArcGIS software, the average weights determined by each group were combined through weighted summation, resulting in three sets of ecosystem sensitivity control area maps (Fig 6). These maps visually represent the distribution of ecosystem sensitivity across different regions and provide strong data support for subsequent comparative analysis and validation efforts.

**Fig 7:**
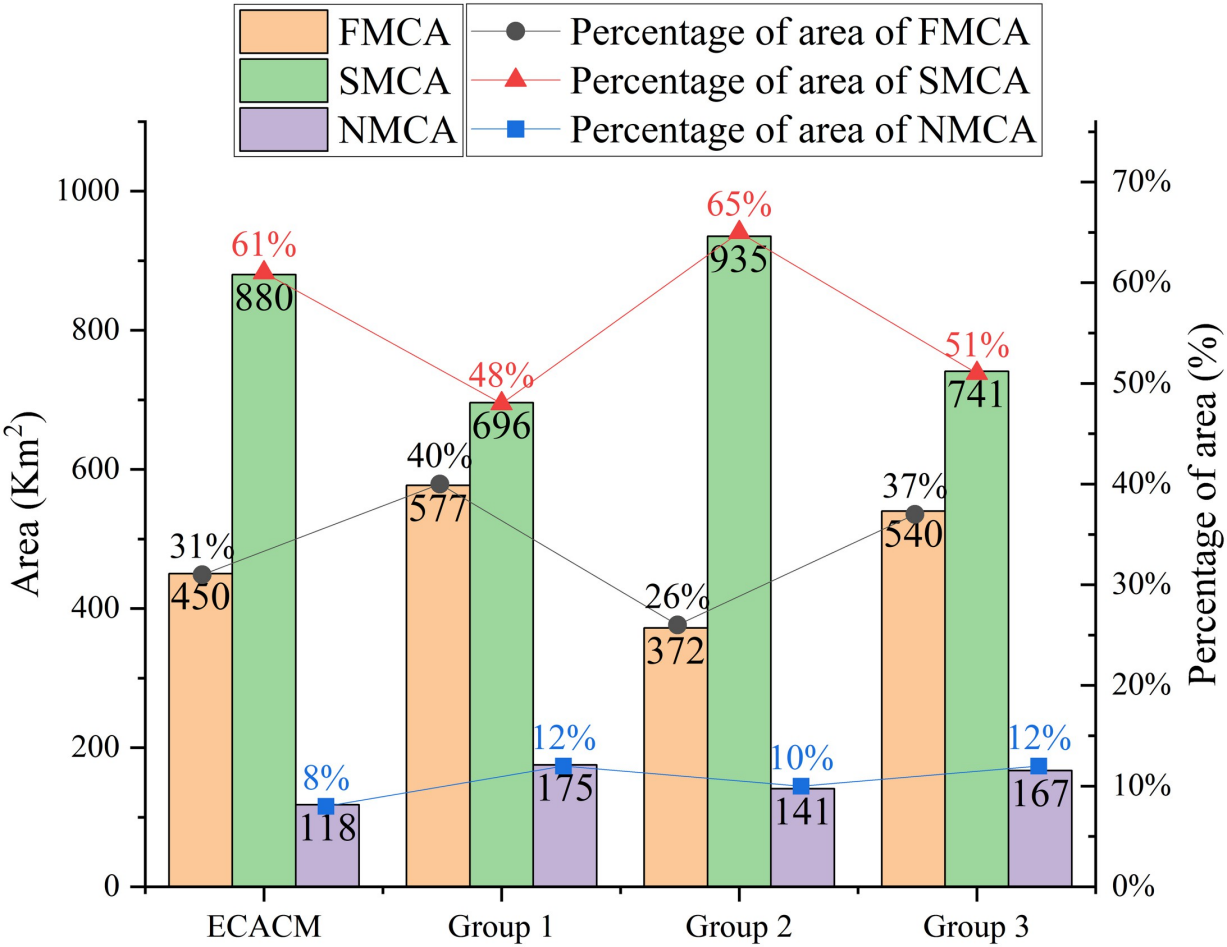
The proportion of control areas in the ESCACM and the three controlled experimental groups.

By comparing the results of the ESCACM with those from the three controlled experimental groups (Fig 7), three conclusions can be drawn. First, significant differences exist in the area proportions of the same control areas among the three experimental groups. For instance, in the second-level management and control areas, the proportion in Group 2 is 17% higher than in Group 1 and 14% higher than in Group 3. Conversely, the proportion of first-level management and control areas in Group 2 is 14% lower than in Group 1 and 11% lower than in Group 3. Additionally, although the non-management and control areas in Groups 1 and 3 exhibit the same proportion, the difference in area between the first-level and second-level management and control areas in Group 1 is 6% smaller than in Group 3. These discrepancies underscore the subjectivity and arbitrariness associated with traditional methods that rely on the AHP, leading to significant variations in the final weightings assigned by different expert groups. Consequently, traditional ecosystem sensitivity classification methods, due to their inherent subjectivity, demonstrate relatively weaker accuracy and objectivity. Second, the area proportions for control areas derived from the ESCACM are more reasonable and accurate. Specifically, while the proportion of non-management and control areas is slightly lower than that in the three controlled experiments, the proportions of first-level and second-level management and control areas within the ESCACM align with the ranges observed in the controlled experiments. Lastly, the proportion of non-management and control areas in the ESCACM is notably lower compared to the three experimental groups. This may result from the specific classification logic used in the raster overlay process of the ESCACM, where areas scoring “3” and “4” are categorized as non-management and control areas. Despite the existence of 27 possible combination methods in the complex overlay operations, only four combinations can ultimately yield a final score of “3” or “4,” contributing to the lower proportion of non-management and control areas. This reduction implies a decrease in construction land, thereby objectively enhancing ecological protection and aligning closely with the goal of promoting sustainable development in urban ecosystems.

### 4.2 Layout strategy for multi-scenario ecosystem sensitivity control area

Balancing the stable development of ecosystems with economic growth is an essential goal for urban development worldwide. Compared to traditional methods of classifying control areas, the core advantage of the ESCACM is its ability to implement precise adjustments and planning strategies for ecosystem sensitivity control areas. These adjustments are tailored to the specific developmental contexts of different regions, government priorities, and planning blueprints, offering a more customized and effective approach.

The multi-scenario ecosystem sensitivity control area layout strategies aim to achieve a win-win situation between ecological protection and economic development. By considering various developmental scenarios and ecosystem characteristics, these strategies enable the creation of more scientifically sound and reasonable ecosystem management plans. They are highly adaptable, allowing for flexible adjustments to layout plans to balance the relationship between ecological protection and economic growth. This adaptability ensures that ecosystems can continue to develop stably, even under the influence of human activities. By establishing different control scenarios, the strategy provides greater flexibility in addressing diverse ecosystem challenges, thereby maintaining ecosystem health and stability.

### 4.3 Scientifically selecting ecosystem sensitivity factors

Assessing the sensitivity of regional ecosystems is a complex and multifaceted process that requires a comprehensive collection of ecological sensitivity factors. However, previous studies have often shown shortcomings in factor selection, frequently choosing only a limited number of factors or including variables with overlapping characteristics. Such approaches can lead to insufficient assessment outcomes, failing to accurately capture the full extent of ecosystem sensitivity in a particular area. In contrast to earlier research, this study adopts a more thorough and detailed method for factor selection, encompassing various dimensions, including topographic and geomorphological factors, ecological and environmental factors, and human activity factors. A total of 18 indicator factors were incorporated to ensure a comprehensive and precise assessment. These factors include conventional data such as elevation, slope, water areas, and land use types, while also considering the unique natural conditions of the Mentougou District and the impacts of mining activities. Moreover, specific and representative factors—such as national nature reserves, scenic areas, soil erosion intensity, soil heavy metals, soil pH alkalinity, and the influence of coal mining—were included to enhance the relevance of the assessment. This meticulous and comprehensive factor system allows for a more integrated and thorough reflection of the ecosystem’s sensitivity in the study area, providing a more scientific and reliable foundation for future ecological protection and management initiatives.

Compared ecosystem management models, the ESCACM offers significant advantages in terms of both accuracy and applicability across different geographical regions and ecological environments. Regarding accuracy, the ESCACM integrates topographic and geomorphological factors, ecological and environmental factors, and human activity factors, conducting a comprehensive evaluation of 18 indicator factors. This approach provides an in-depth and holistic assessment of the ecological environment, thereby ensuring precise ecosystem sensitivity zoning. In terms of adaptability, the multi-scenario layout strategy for ecosystem sensitivity management areas allows for flexible adjustments by considering various developmental scenarios and ecosystem characteristics.

### 4.4 Implementing ESCACM: Potential challenges, barriers, and countermeasures

The ESCACM encounters various potential challenges and barriers, which may originate from multiple levels, including legal policies, conflicts of economic interests, public awareness, and funding support.

1. Legal Policies: The legal and regulatory framework governing ecosystem sensitivity control areas in China is still underdeveloped, resulting in a lack of clear legal foundations and mandatory safeguards for implementing related policies.
2. Economic Interest Conflicts: The introduction of the ESCACM may restrict the development and expansion of certain industries, which could lead to reduced local employment opportunities and decreased fiscal revenue, ultimately impacting regional economic growth.
3. Public Awareness: Some segments of the public may lack a sufficient understanding of ecological and environmental protection, leading to low levels of support and participation in the ESCACM process.
4. Funding Support: Implementing the ESCACM necessitates significant long-term financial investment in ecological protection initiatives, and funding shortages often pose a substantial constraint on project advancement.

To tackle these potential challenges and barriers, collaboration among governments, research institutions, non-governmental organizations, and local communities is essential. Effective measures should be actively implemented to identify and implement solutions. The following specific recommendations are proposed:

1. **Legal Policies:** Enhance the relevant laws and regulations governing ecosystem sensitivity control areas to establish a more robust legal framework.
2. **Economic Interest Conflicts:** Develop a scientifically sound ecological compensation mechanism that encourages stakeholders—including enterprises, local governments, and community residents—to participate in the decision-making process. Facilitating dialogue among different interest groups will help ensure their voices are heard and promote consensus through negotiation to strike a balance of interests.
3. **Public Awareness:** Strengthen mechanisms for public participation and implement educational campaigns to raise environmental awareness and encourage greater public involvement in ecological initiatives.
4. **Funding Support:** Obtain necessary financial resources through government grants and subsidies, engage financial institutions, pursue social investments, and seek support from charitable funds.

### 4.5 Limitations and future work

This study centers on weight allocation methods for indicator factors and introduces the ESCACM to address the subjectivity and arbitrariness associated with traditional classification methods, such as the AHP. However, there are notable limitations. When aggregating individual ecosystem factors into a multi-factor comprehensive sensitivity assessment, the lack of quantitative data to describe these factors and the inability to directly assign weights based on intrinsic relationships necessitate the use of subjective weighting methods like AHP. This reliance introduces a degree of subjectivity in the weight allocation for individual ecosystem factors. Therefore, future research should focus on developing methods to ensure that weight allocation results are more objective.

Additionally, with the rapid advancement of technology, the management of ecosystem sensitivity zoning is poised to encounter new opportunities. The integration of cutting-edge technologies such as remote sensing, machine learning, and big data is expected to enhance the accuracy and efficiency of ecosystem sensitivity management significantly.

Remote sensing serves as a powerful tool that enables real-time tracking of subtle changes in ecosystems, such as fluctuations in vegetation cover density and alterations in the extent of water bodies. It provides valuable real-time monitoring data essential for the dynamic regulation of sensitive ecological areas.

Machine learning, known for its robust data processing capabilities, can analyze vast datasets to extract meaningful information for precise predictions and classifications. When applied to remote sensing data, machine learning can effectively identify features closely related to ecosystem sensitivity, thus offering robust support for in-depth research.

Furthermore, big data analytics excels in the rapid processing and examination of large datasets, revealing patterns and trends associated with ecological changes and uncovering deeper causes behind ecosystem shifts. It can effectively monitor and evaluate the effectiveness of ecosystem sensitivity zoning policies, providing scientific evidence to inform governmental decision-making and facilitating the precise adjustment and optimization of these policies.

## 5 Conclusions

This study examines the Mentougou District and introduces the ESCACM, which categorizes the region into three zones: FMCA, SMCA, and NMCA. The conclusions of this study can be summarized as follows:

1. The ESCACM achieves theoretical dimensionality reduction for relevant relational factors, significantly enhancing the weight of quantitative data in the calculation process, thereby ensuring the objectivity and scientific validity of the results.
2. The ESCACM provides three distinct multi-scenario ecosystem sensitivity zoning strategies, allowing for flexible adjustments to the layout based on actual conditions, promoting the harmonious coexistence of ecological protection and economic development while maintaining ecosystem health and stability.
3. According to the ESCACM results, the FMCA is primarily concentrated in the western and central regions of the study area, covering a total area of 450 km^2^, which is approximately 31% of the total area. The SMCA is mainly found in the central part of the study area, with a more concentrated distribution totaling 880 km^2^, or about 61% of the total area. The NMCA is predominantly located in the eastern urban area and along the central ring road, with a total area of 118 km^2^, accounting for roughly 8% of the study area.
4. Compared to the experimental results from three traditional methods, the area designated as NMCA by the ESCACM is 2% smaller, indicating a further reduction in construction land area, with a corresponding increase in the area allocated for ecological protection.

Overall, the ESCACM offers a more scientific and flexible approach to ecosystem sensitivity zoning, providing a valuable framework for future ecological protection planning.

## Supporting information

S1 Fig Sensitivity analysis for each individual factor within the topographic and geomorphological category. (DOCX)

S2 Fig Sensitivity analysis for each individual factor within the human activity category. (DOCX)

S1 Table Discriminant matrix for each single factor of topographic and geomorphological factors. (DOCX)

S2 Table Discriminant matrix for each single factor of ecological and environmental factors. (DOCX)

S3 Table Discriminant matrix for each single factor of human activities factors. (DOCX)

S4 Table Detailed table of expert empowerment for the three control experiments. (DOCX)
