# Supplementary figures and images for: An investigation into the zoning of ecosystem sensitivity control areas in Mentougou District (Beijing, China)
Source: PLoS One. 2024 Dec 19;19(12):e0316025. doi: 10.1371/journal.pone.0316025 (PMC11658590; doi:10.1371/journal.pone.0316025)

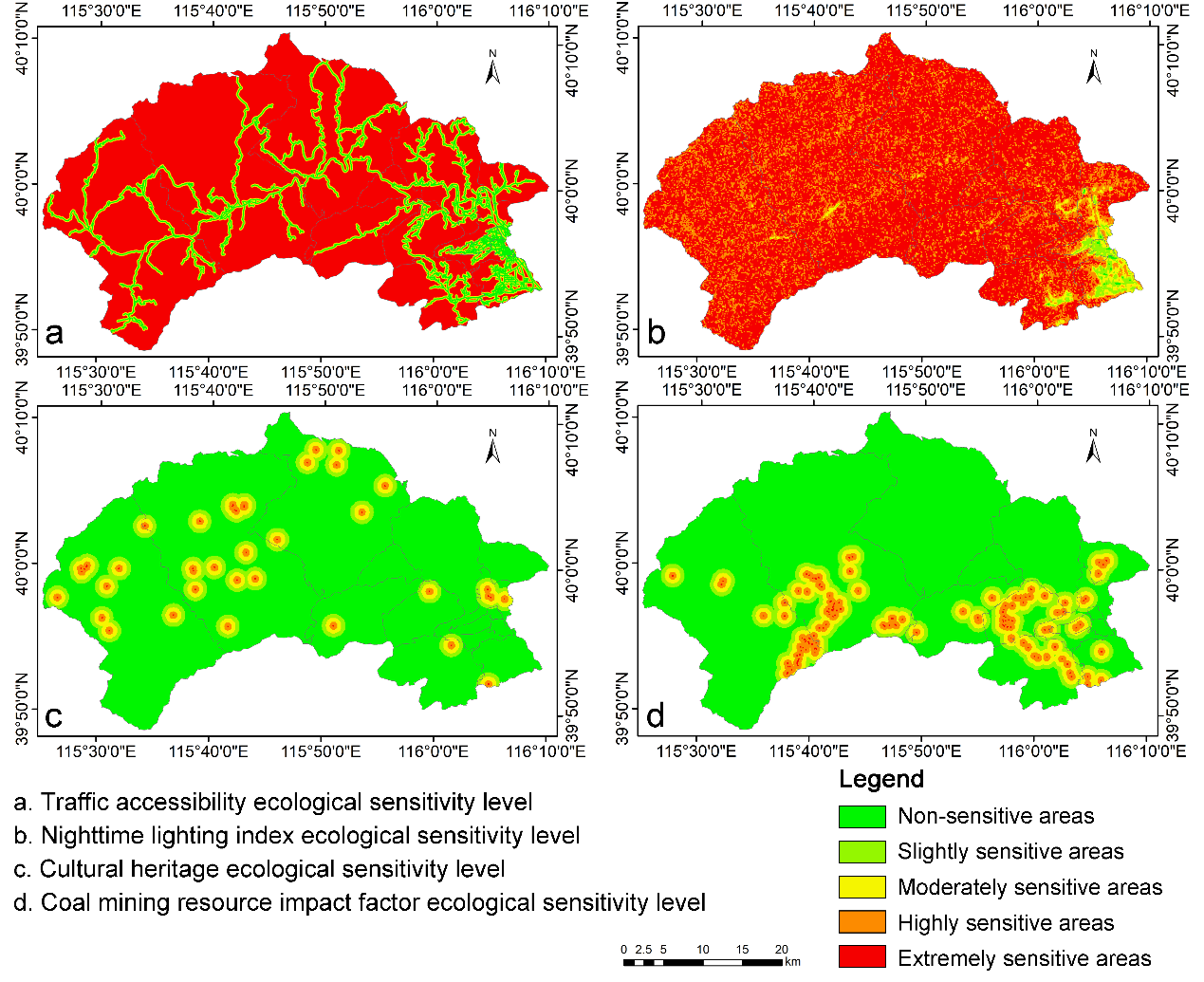


**S2 Fig. Sensitivity analysis for each individual factor within the human activity category.**

Supplement: S2 Fig — (DOCX) [file pone.0316025.s002.docx]
