# Supplementary material for: An investigation into the zoning of ecosystem sensitivity control areas in Mentougou District (Beijing, China)
Source: PLoS One. 2024 Dec 19;19(12):e0316025. doi: 10.1371/journal.pone.0316025 (PMC11658590; doi:10.1371/journal.pone.0316025)
Supplement: S1 Table — (DOCX) [file pone.0316025.s003.docx]

**S1 Table. Discriminant matrix for each single factor of topographic and geomorphological factors.**

|  | **Elevation** | **Slope** | **Slope aspect** | **Relief degree of land surface** | **Water areas** | **Water source protection areas** | **Weight** | **Consistency test** |
| --- | --- | --- | --- | --- | --- | --- | --- | --- |
| **Elevation** | 1 | 3 | 3 | 3 | 3 | 3 | 0.3528 | CR=0.0799  δmax=6.5033 |
| **Slope** | 1/3 | 1 | 5 | 3 | 1 | 1 | 0.1838 |  |
| **Slope aspect** | 1/3 | 1/5 | 1 | 3 | 1/3 | 1/3 | 0.0790 |  |
| **Relief degree of land surface** | 1/3 | 1/3 | 1/3 | 1 | 1/3 | 1/3 | 0.0571 |  |
| **Water areas** | 1/3 | 1 | 3 | 3 | 1 | 1/2 | 0.1455 |  |
| **Water source protection areas** | 1/3 | 1 | 3 | 3 | 2 | 1 | 0.1818 |  |

Note: The obtained *CR*=0.0799. Since *CR<*0.1, this judgment matrix satisfies the consistency test. The weights of elevation, slope, slope aspect, relief degree of the land surface, water areas, and water source protection areas were determined as 0.3528, 0.1838, 0.0790, 0.0571, 0.1455, and 0.1818, respectively.
