# Supplementary material for: An investigation into the zoning of ecosystem sensitivity control areas in Mentougou District (Beijing, China)
Source: PLoS One. 2024 Dec 19;19(12):e0316025. doi: 10.1371/journal.pone.0316025 (PMC11658590; doi:10.1371/journal.pone.0316025)
Supplement: S2 Table — (DOCX) [file pone.0316025.s004.docx]

**S2 Table. Discriminant matrix for each single factor of ecological and environmental factors.**

|  | **NDVI** | **Land use types** | **Precipitation** | **Soil erosion intensity** | **Soil heavy metals** | **Soil PH acidity** | **National nature reserve** | **Scenic areas** | **Weight** | **Consistency test** |
| --- | --- | --- | --- | --- | --- | --- | --- | --- | --- | --- |
| **NDVI** | 1 | 1/3 | 5 | 7 | 7 | 7 | 3 | 7 | 0.2704 | CR=0.0755  δmax=8.7454 |
| **Land use types** | 3 | 1 | 5 | 5 | 5 | 5 | 3 | 7 | 0.3355 |  |
| **Precipitation** | 1/5 | 1/5 | 1 | 1 | 3 | 3 | 1/5 | 1/3 | 0.0519 |  |
| **Soil erosion intensity** | 1/7 | 1/5 | 1 | 1 | 1 | 1 | 1/5 | 1/3 | 0.0360 |  |
| **Soil heavy metals** | 1/7 | 1/5 | 1/3 | 1 | 1 | 1 | 1/7 | 1/3 | 0.0309 |  |
| **Soil pH alkalinity** | 1/7 | 1/5 | 1/3 | 1 | 1 | 1 | 1/7 | 1/3 | 0.0309 |  |
| **National nature reserve** | 1/3 | 1/3 | 5 | 5 | 7 | 7 | 1 | 3 | 0.1682 |  |
| **Scenic areas** | 1/7 | 1/7 | 3 | 3 | 3 | 3 | 1/3 | 1 | 0.0764 |  |

Note: The obtained *CR*=0.0755. Since *CR<*0.1, this judgment matrix satisfies the consistency test and allows to determine the weights of NDVI, land use types, precipitation, soil erosion intensity, soil heavy metals, soil pH alkalinity, national nature reserves, and scenic areas as 0.2704, 0.3355, 0.0519, 0.0360, 0.0309, 0.0309, 0.1682, and 0.0764, respectively.
