# Supplementary material for: An investigation into the zoning of ecosystem sensitivity control areas in Mentougou District (Beijing, China)
Source: PLoS One. 2024 Dec 19;19(12):e0316025. doi: 10.1371/journal.pone.0316025 (PMC11658590; doi:10.1371/journal.pone.0316025)
Supplement: S3 Table — (DOCX) [file pone.0316025.s005.docx]

**S3 Table. Discriminant matrix for each single factor of human activities factors.**

|  | **Traffic accessibility** | **Nighttime light index** | **Cultural heritage** | **Coal mining resource impact factors** | **Weight** | **Consistency test** |
| --- | --- | --- | --- | --- | --- | --- |
| **Traffic accessibility** | 1 | 3 | 3 | 5 | 0.5048 | CR=0.0742  δmax=4.1981 |
| **Nighttime light index** | 1/3 | 1 | 1/3 | 3 | 0.1431 |  |
| **Cultural heritage** | 1/3 | 3 | 1 | 5 | 0.2876 |  |
| **Coal mining resource impact factors** | 1/5 | 1/3 | 1/5 | 1 | 0.0645 |  |

Note: The obtained *CR*=0.0742. Since *CR<*0.1, this judgment matrix satisfies the consistency test and allows the determination of the weights of traffic accessibility, the nighttime light index, cultural heritage, and coal mining resource impact factors to be 0.5048, 0.1431,0.2876, and 0.0645, respectively.
