# Supplementary material for: An investigation into the zoning of ecosystem sensitivity control areas in Mentougou District (Beijing, China)
Source: PLoS One. 2024 Dec 19;19(12):e0316025. doi: 10.1371/journal.pone.0316025 (PMC11658590; doi:10.1371/journal.pone.0316025)
Supplement: S4 Table — (DOCX) [file pone.0316025.s006.docx]

**S4 Table. Detailed table of expert empowerment for the three control experiments.**

| **Expert serial number** | **Weighting of topographic and geomorphological factors** | **Weighting of ecological and environmental factors** | **Weighting of human activity factors** | **CR** | **δmax** |
| --- | --- | --- | --- | --- | --- |
| **Expert 01** | 0.6144 | 0.2684 | 0.1172 | 0.0707 | 3.0735 |
| **Expert 02** | 0.4286 | 0.1429 | 0.4286 | 0 | 3 |
| **Expert 03** | 0.4286 | 0.4286 | 0.1429 | 0 | 3 |
| **Expert 04** | 0.7306 | 0.1884 | 0.0810 | 0.0624 | 3.0649 |
| **Expert 05** | 0.2000 | 0.2000 | 0.6000 | 0 | 3 |
| **Expert 06** | 0.2583 | 0.6370 | 0.1047 | 0.037 | 3.0385 |
| **Expert 07** | 0.2583 | 0.6370 | 0.1047 | 0.037 | 3.0385 |
| **Expert 08** | 0.4286 | 0.4286 | 0.1429 | 0 | 3 |
| **Expert 09** | 0.2797 | 0.6267 | 0.0936 | 0.0825 | 3.0858 |
| **Expert 10** | 0.2797 | 0.6267 | 0.0936 | 0.0825 | 3.0858 |
| **Expert 11** | 0.4286 | 0.1429 | 0.4286 | 0 | 3 |
| **Expert 12** | 0.6144 | 0.2684 | 0.1172 | 0.0707 | 3.0735 |
| **Expert 13** | 0.2684 | 0.6144 | 0.1172 | 0.707 | 3.0735 |
| **Expert 14** | 0.4286 | 0.1429 | 0.4286 | 0 | 3 |
| **Expert 15** | 0.1007 | 0.6738 | 0.2255 | 0.0825 | 3.0858 |
| **Average weight of Group 1** | 0.4804 | 0.2457 | 0.2739 | - | - |
| **Average weight of Group 2** | 0.3009 | 0.5912 | 0.1079 | - | - |
| **Average weight of Group 3** | 0.3681 | 0.3685 | 0.2634 | - | - |
